# Penicillin-Binding Protein Occupancy Dataset for 18 β-Lactams and 4 β-Lactamase Inhibitors in Neisseria gonorrhoeae

**DOI:** 10.1128/spectrum.00692-23

**Published:** 2023-04-24

**Authors:** Silvia López-Argüello, Maria Montaner, Amanda Mármol-Salvador, Ana Velázquez-Escudero, Fernando Docobo-Pérez, Antonio Oliver, Bartolome Moya

**Affiliations:** a Servicio de Microbiología and Unidad de Investigación, Hospital Universitario Son Espases, Health Research Institute of the Balearic Islands (IdISBa), Palma, Spain; b Departamento de Microbiología, Facultad de Medicina, Universidad de Sevilla, Seville, Spain; c Instituto de Biomedicina de Sevilla, Hospital Universitario Virgen Macarena/CSIC/Universidad de Sevilla, Seville, Spain; d Centro de Investigación Biomédica en Red en Enfermedades Infecciosas (CIBERINFEC), Madrid, Spain; Universidad de Buenos Aires

**Keywords:** penicillin-binding proteins (PBP), *N. gonorrhoeae*, gonococcus, β-lactams, β-lactam resistance

## Abstract

The lack of effective first-line antibiotic treatments against Neisseria gonorrhoeae, and the worldwide dissemination of resistant strains, are the main drivers of a worsening global health crisis. β-lactam antibiotics have been the backbone of therapeutic armamentarium against gonococci. However, we are lacking critical insights to design rationally optimized therapies. In the present work, we generated the first PBP-binding data set on 18 currently available and clinically relevant β-lactams and 4 β-lactamase inhibitors in two N. gonorrhoeae ATCC type collection strains, 19424 and 49226 (PBP2 type XXII and A39T change in *mtrR*). PBP binding (IC_50_) was determined via the Bocillin FL binding assay in isolated membrane preparations. Three clusters of differential PBP IC_50_s were identified and were mostly consistent across both strains, but with quantitative differences. Carbapenems were coselective for PBP2 and PBP3 (0.01 to 0.03 mg/L). Third- and fourth-generation cephalosporins cefixime, cefotaxime, ceftazidime, cefepime, and ceftriaxone showed the lowest IC_50_ values for PBP2 (0.01 mg/L), whereas cefoxitin, ceftaroline, and ceftolozane required higher concentrations (0.04 to >2 mg/L). Aztreonam was selective for PBP2 in both strains (0.03 to 0.07 mg/L); amdinocillin bound this PBP at higher concentrations (1.33 to 2.94 mg/L). Penicillins specifically targeted PBP2 in strain ATCC 19424 (0.02 to 0.19 mg/L) and showed limited inhibition in strain ATCC 49226 (0.01 to >2 mg/L). Preferential PBP2 binding was observed by β-lactam-based β-lactamase inhibitors sulbactam and tazobactam (1.07 to 6.02 mg/L); meanwhile, diazabicyclooctane inhibitors relebactam and avibactam were selective for PBP3 (1.27 to 5.40 mg/L). This data set will set the bar for future studies that will help the rational use and translational development of antibiotics against multidrug-resistant (MDR) N. gonorrhoeae.

**IMPORTANCE** The manuscript represents the first N. gonorrhoeae PBP-binding data set for 22 chemically different drugs in two type strains with different genetic background. We have identified three clusters of drugs according to their PBP binding IC_50_s and highlighted the binding differences across the two strains studied. With the currently available genomic information and the PBP-binding data, we have been able to correlate the target attainment differences and the mutations that affect the drug uptake with the MIC changes. The results of the current work will allow us to develop molecular tools of great practical use for the study and the design of new rationally designed therapies capable of combating the growing MDR gonococci threat.

## INTRODUCTION

Sexually transmitted infections (STIs) are a major public health problem worldwide affecting quality of life and causing serious morbidity and mortality. Gonorrhea, one of the oldest recorded human diseases, is an STI caused by Neisseria gonorrhoeae (gonococcus), a facultative anaerobic, immobile, and nonencapsulated Gram-negative diplococcus. The obligate human pathogen N. gonorrhoeae is a significant global public health problem of growing magnitude with more than 106 million new cases being diagnosed every year worldwide (1). Without an effective vaccine, the lack of effective treatment for controlling gonorrhea has caused N. gonorrhoeae to be classified as an urgent public health threat globally. In fact, the Centers for Disease Control (CDC) classified N. gonorrhoeae as a “superbug,” and the World Health Organization (WHO) classified it as a “Priority 2” microorganism, announcing a near future in which gonorrhea would become untreatable (2 to 4).

Due to single dose therapy, successful horizontal gene transfer, genome plasticity, and rare resistance-derived fitness cost, N. gonorrhoeae has developed and retained resistance to every major antibiotic class, matching every historical milestone in antibiotic discovery. Worldwide emergence of gonococcal strains exhibiting multidrug resistance (MDR) and extensive drug resistance (XDR) is of great concern (5 to 8). In fact, as of 2012 cefixime is no longer recommended, and in many settings worldwide (with the current azithromycin resistance rates), ceftriaxone (single high dose) is the last remaining option for empirical first-line antimicrobial therapy (9 to 12).

Particularly, gonococci resistance determinants that affect current first-line treatments include (i) plasmid-mediated high-level resistance to penicillins determined by plasmids harboring TEM-1-type β-lactamases (13, 14); (ii) chromosomally mediated penicillin and extended spectrum cephalosporins (ESCs) resistance due to mutations affecting penicillin-binding proteins (PBPs) targets, frequently in the *penA* gene encoding PBP2, the main gonococcal lethal target for β-lactam antimicrobials (from single point mutations near the active site to mosaic *penA* genes that contain up to 60 to 70 amino acid changes) (5, 8, 11, 14 to 16); (iii) overexpression of the MtrCDE efflux pump conferring diminished sensitivity to hydrophobic antimicrobials, such as macrolides, β-lactams, ciprofloxacin, and tetracycline (17); (iv) mutations in outer membrane protein (OMP) PorB1b (*penB* determinant) associated with a decreased susceptibility to penicillin, cephalosporins, and tetracyclines (18, 19); and (v) reduced affinity for the 50S ribosomal macrolide target (23S rRNA SNPs) together with *mtrR* mutations, which constitute the macrolides’ main resistance determinants (5, 17, 20 to 22).

As a result of multiple resistance mechanisms coexisting in successful lineages, antimicrobial resistance rates and gonococcal treatment failures have increased worldwide (5, 7, 11, 23 to 27). Thus, nonoptimized dual-antimicrobial regimens might not be effective long-term solutions. Furthermore, the preclinical pipeline remains virtually empty of agents targeted to clinical development for N. gonorrhoeae treatment (10, 28 to 31).

During the last 80 years, β-lactams have recurrently been the cornerstone of our therapeutic arsenal against N. gonorrhoeae infections. However, the shortage of new antimicrobial compounds and the lack of mechanistic support of empirical combinations to treat resistant gonococcal isolates could be devastating for STIs’ antibiotic stewardship (9 to 11, 32 to 34).

The genome of N. gonorrhoeae encodes 5 PBPs. The two low molecular mass class C PBPs, PBP3 (*dacB*) and 4 (*pbpG*) (which catalyze carboxypeptidase and endopeptidase activity), are nonessential for cell viability but do play a role in cell morphology maintenance (35, 36). A third low molecular mass PBP with carboxypeptidase activity, *dacC* (not identified in labeled penicillin binding assays), has been also described. However, its binding ability and role in the intrinsic β-lactam resistance are still unknown (36, 37). The two high molecular mass transpeptidases, PBP1 (Class A) and PBP2 (Class B) are both essential. Gonococcal PBP1 (*ponA*) is the homolog of Escherichia coli PBP1a (*mrcA*), responsible for transglycosylation and transpeptidation, and participates in cell growth and elongation, while PBP2 (*penA*) is the homolog of *E.coli* PBP3 (*ftsI*), an essential peptidoglycan transpeptidase that catalyzes the cross-linking of peptidoglycan-adjacent strand functions during cell wall synthesis and division (38). PBP2, the primary clinical target, is inhibited at 10-fold lower concentrations than PBP1 in β-lactam-susceptible strains (5, 15, 16). PBP3 (*dacB*) is homologous to E. coli PBP4 (*dacB*), while PBP4 (*pbpG*) is most similar to E. coli PBP7 (*pbpG*) (35, 39).

Although the gonococcal PBP inhibitory concentrations of cephalosporins have been thoroughly characterized for the primary target PBP2 (33), we are not aware of any published PBP-binding IC_50_s data set in N. gonorrhoeae, and PBP binding profiles are known for roughly a few β-lactams (38, 40, 41). Therefore, the scientific basis to enhance β-lactam-based therapy to inactivate PBPs more efficiently is missing for this important pathogen (42).

In the present work, we aimed to characterize the PBP binding profiles for 18 currently available and clinically relevant β-lactams and four β-lactamase inhibitors in two N. gonorrhoeae type collection strains, ATCC 19424 and ATCC 49226. Our PBP binding data set for a comprehensive set of chemically diverse β-lactam antibiotics and β-lactamase inhibitors will help us rank order the drugs according to their PBP selectivity and rationally design new compounds and combinations. Our results warrant future research to rationally optimize β-lactam-based combination antibiotic therapy against resistant isolates.

## RESULTS

**FIG 1:**
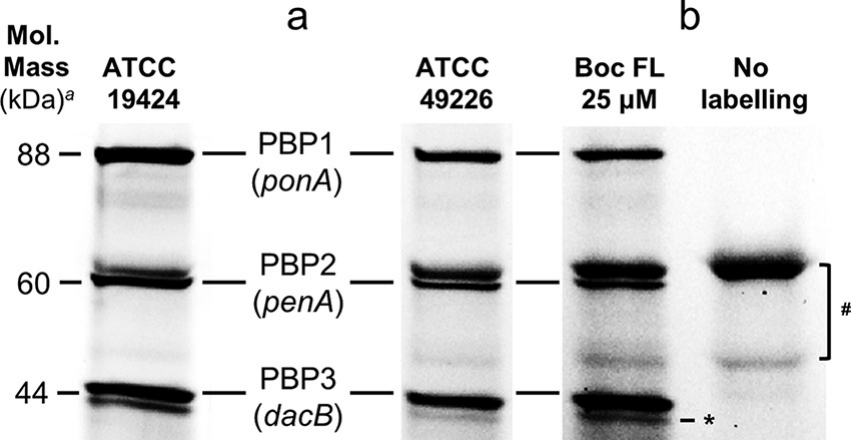
Penicillin-binding protein profiles of N. gonorrhoeae strains ATCC 19424 and ATCC 49226. PBPs were labeled with Bocillin FL, separated by SDS-PAGE, and quantified via the ImageQuantTL program. *^a^*Apparent molecular mass relative to Precision Plus Protein Dual Color Standards (range 10 to 250 kDa) (Bio-Rad Laboratories, Hercules, CA). (a) Comparison of the PBP profiles of the two studied N. gonorrhoeae strains. (b) Membrane preparation loaded with and without Bocillin FL labelling. ^#^Two autofluorescence bands were present in both strains with and without Bocillin FL labelling (not bound by any of the drugs tested); they were excluded from any further analysis. *The band below PBP3 is a potential proteolytic band and was excluded from further analysis.

Membrane fractions from N. gonorrhoeae ATCC 19424 and ATCC 49226 were labeled with Bocillin FL for 30 min. We were able to identify PBP1, 2, and 3, which had the same apparent molecular mass in both strains (Fig. 1A). In accordance with previous works using radiolabeled penicillin, we were not able to detect *dacC* with our experimental approach (40). Stefanova et al. showed (not as evident as PBP1 to 3) iodopenicillin (^125^I) binding to PBP4 (*pbpG*), though it was not visible in our assay (35). Two autofluorescence bands (no binding to β-lactams) and one potential proteolytic artifact without apparent antibiotic binding properties were observed with no interference with our analysis (Fig. 1B) (48). None of the ATCC strains expresses a β-lactamase; however, they possess different allelic variants for the genes intimately related with β-lactam resistance: PBPs, porin PorB, and efflux pump regulator *mrtR* (Table 1). Both strains possess the *porB1a* porin allele, which has been correlated with lower MICs.

**TABLE 1:** Amino acid changes and allelic variants of N. gonorrhoeae ATCC 19424 and ATCC 49226

Strain^*a*^	Mutations^*b*^						
	ST (NG-MAST)	PBP1 (*ponA*)	PBP2 (*penA*)		PBP3 (*dacB*)	PorB1a (*penB)*	*mtrR*
			Allelic changes	Type (mosaic)			
ATCC 19424	266	WT	H541N	XV (no)	T252S, H278Q, S285A, I364M	A121G	WT
ATCC 49226	1572	A375T, F666S	R345_D346insD, F504L, A505V, A516G, H541N, P552V, K555Q, I556V, I566V, T573_A574insN, A574V	XXII (no)	T252S, H278Q, S285A, I364M	G120D, A121G	A39T

a The sequences and complete genomes for the N. gonorrhoeae ATCC strains 19424 and 49226 were obtained from the ATCC (American Type Culture Collection) genome portal.

b The wild-type PBP profiles from N. gonorrhoeae were obtained from the following: PBP1 from strain FA19 (GenBank accession number U72876), PBP2 from strain LM306 (GenBank accession number M32091), PBP3 from strain FA1090 (GenBank accession number AF071224), and *mtrR* from strain FA19 (GenBank accession number CP012026.1). Alignments were performed using the blastp suite (protein-protein BLAST).

**FIG 2:**
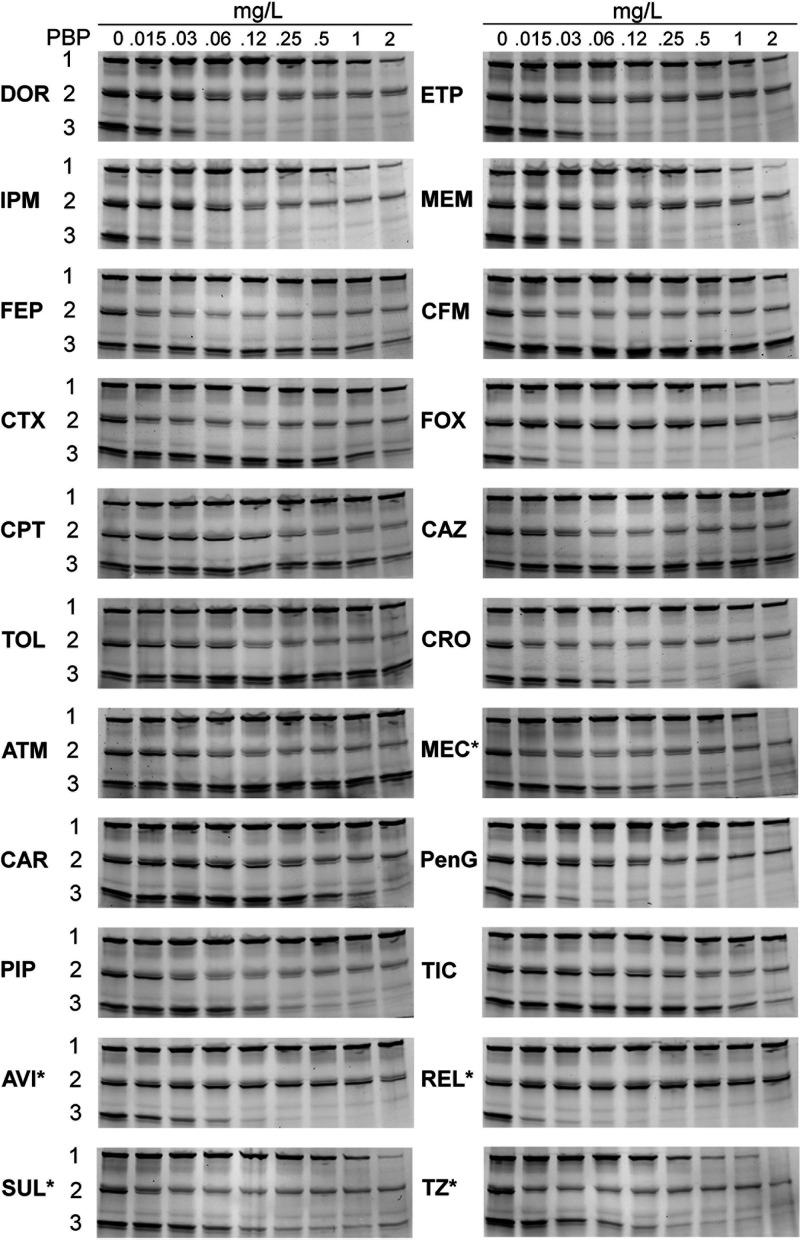
Binding patterns of β-lactams in N. gonorrhoeae PBPs from strain ATCC 19424. DOR, doripenem; ETP, ertapenem; IPM, imipenem; MEM, meropenem; FEP, cefepime; CFM, cefixime; CTX, cefotaxime; FOX, cefoxitin; CPT, ceftaroline; CAZ, ceftazidime; TOL, ceftolozane; CRO, ceftriaxone; ATM, aztreonam; MEC, amdinocillin (amdinocillin); CAR, carbenicillin; PenG, penicillin G; PIP, piperacillin; TIC, ticarcillin; AVI, avibactam; REL, relebactam; SUL, sulbactam; TZ, tazobactam. The membrane preparations were incubated with the indicated β-lactams for 30 min before Bocillin FL labeling. Labeled PBPs were separated by SDS-PAGE and detected using a fluorimager. The range of concentrations tested was 0.015 to 2 mg/L. *MEC and BLIs studied ranged from 2 to 256 mg/L.

**FIG 3:**
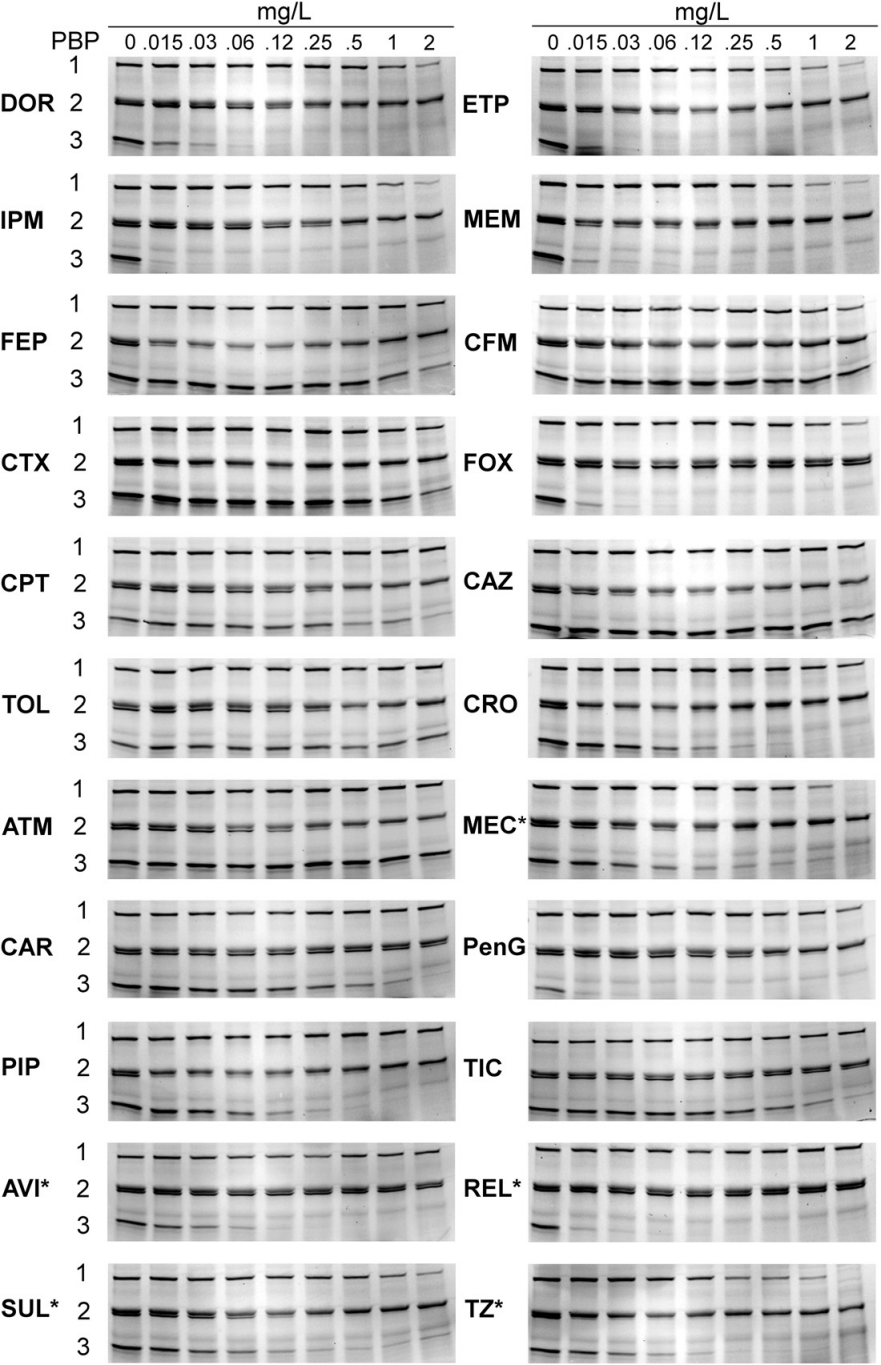
Binding patterns of β-lactams for N. gonorrhoeae PBPs from strain ATCC 49226. The membrane preparations were incubated with the indicated β-lactams for 30 min before Bocillin FL labeling. The range of concentrations tested was 0.015 to 2 mg/L. Please see Fig. 2 for abbreviations. *MEC and BLIs studied ranged from 2 to 256 mg/L.

We generated the binding profiles and inhibition concentrations (IC_50_s) of 18 different β-lactams and four β-lactamase inhibitors in triplicate for two wild-type reference N. gonorrhoeae strains, ATCC 19424 (Fig. 2; Fig. S1 in the supplemental material) and ATCC 49226 (Fig. 3; Fig. S2).

A drug was considered selective for a PBP if the IC_50_ was at least 4-fold lower than that of the next most inhibited PBP (Table S1) (49). If any additional PBP binding was below the 4-fold threshold, the compound was then considered coselective (50, 51). The MIC/min IC_50_ parameter (Table S1) represents the ratio of the MIC to the minimum IC_50_ value. Higher ratios indicate lower antibacterial potency and may correlate with a lower therapeutic efficacy. Drugs with minimum IC_50_ values for PBP3 displayed the higher MIC values.

In both strains, all carbapenems but imipenem were coselective for PBP2 and PBP3 (i.e., Bocillin FL signal reduction occurred at very low carbapenem concentrations) with overall very low IC_50_s (Table 2; Table S2) (52). The carbapenems IC_50_ for PBP3 were the lowest among all the drugs tested (0.01 to 0.03 mg/L). Ertapenem was the carbapenem with the lowest IC_50_. The MICs for the carbapenems ranged from 0.004 mg/L to 0.032 mg/L in the two ATCC strains, with the exception of imipenem. Imipenem showed the highest MIC (0.032 to 0.064 mg/L) and IC_50_ (PBP2 = 0.07 to 0.17 mg/L) values for all the targets.

**TABLE 2:** PBP IC_50_ and MICs of β-lactam antibiotics and BLIs in N. gonorrhoeae ATCC 19424 and ATCC 49226

Strain^*a*^	IC_50_ and MIC of the indicated drug (mg/L)^*b*^										
	DOR	ETP	IPM	MEM	FEP	CFM	CTX	FOX	CPT	CAZ	TOL
ATCC 19424											
PBP1	1.13 ± 0.21	0.48 ± 0.22	0.4 ± 0.24	0.24 ± 0.07	>2	0.92 ± 0.28	1.85 ± 0.24	0.58 ± 0.1	>2	>2	>2
PBP2	0.06 ± 0.03	0.04 ± 0.02	0.07 ± 0.02	0.03 ± 0.00	0.01 ± 0.00	0.01 ± 0	0.01 ± 0.00	0.39 ± 0.05	0.15 ± 0.09	0.01 ± 0.00	0.04 ± 0.01
PBP3	0.02 ± 0.00	0.03 ± 0.01	0.01 ± 0.00	0.02 ± 0.00	1.57 ± 0.42	>2	1.43 ± 0.63	0.01 ± 0.00	1.9 ± 1.0	>2	>2
*MIC*	*0.004*	*0.004*	*0.032*	*0.008*	*0.008*	*0.004*	*0.004*	*0.25*	*0.032*	*0.016*	*0.032*
ATCC 49226											
PBP1	1.08 ± 0.22	0.57 ± 0.06	0.8 ± 0.56	0.45 ± 0.21	>2	>2	>2	0.91 ± 0.02	>2	>2	>2
PBP2	0.08 ± 0.03	0.01 ± 0.00	0.17 ± 0.08	0.02 ± 0.01	0.01 ± 0.00	0.02 ± 0.00	0.01 ± 0.00	>2	0.21 ± 0.01	0.01 ± 0.00	0.27 ± 0.09
PBP3	0.02 ± 0.01	0.01 ± 0.00	0.01 ± 0.00	0.01 ± 0.00	1.18 ± 0.38	>2	1.02 ± 0.34	0.01 ± 0.00	1.29 ± 0.03	>2	>2
*MIC*	*0.032*	*0.008*	*0.064*	*0.016*	*0.032*	*0.016*	*0.008*	*0.5*	*0.5*	*0.032*	*0.250*
	CRO	ATM	MEC^*c*^	CAR	PenG	PIP	TIC	AVI^*c*^	REL^*c*^	SUL^*c*^	TZ^*c*^
ATCC 19424											
PBP1	1.87 ± 0.53	>2	102 ± 2.26	>2	1.89 ± 0.39	>2	>2	>512	>512	55 ± 17	19 ± 11
PBP2	0.01 ± 0.00	0.03 ± 0.01	1.33 ± 0.15	0.13 ± 0.04	0.05 ± 0.02	0.02 ± 0.00	0.19 ± 0.14	117 ± 7.22	>512	1.18 ± 0.04	1.07 ± 0.02
PBP3	0.07 ± 0.02	>2	11.92 ± 1.47	0.57 ± 0.13	0.02 ± 0.01	0.11 ± 0.04	1.05 ± 0.22	2.33 ± 1.12	1.27 ± 0.12	9.31 ± 4.17	4.72 ± 1.81
*MIC*	*0.004*	*0.032*	*1*	*0.032*	*0.064*	*0.001*	*0.016*	*128*	>*256*	*0.25*	*0.125*
ATCC 49226											
PBP1	1.9 ± 0.19	>2	155	155 ± 24	>2	>2	>2	>256	>256	203 ± 118	25 ± 6.94
PBP2	0.01 ± 0.00	0.07 ± 0.03	5.88	5.88 ± 1.94	0.26 ± 0.08	0.01 ± 0.00	>2	>256	>256	6.02 ± 0.34	1.44 ± 0.05
PBP3	0.07 ± 0.02	>2	9.23	9.23 ± 3.52	0.01 ± 0.00	0.09 ± 0.02	0.63 ± 0.10	5.40 ± 1.87	1.31 ± 0.26	6.36 ± 0.21	5.69 ± 0.51
*MIC*	*0.002*	*0.125*	*8*	*0.5*	*0.125*	*0.125*	*0.5*	>*256*	>*256*	*4*	*1*
	PIP/TZ^*d*^				CAZ/AVI^*d*^				TOL/TZ^*d*^		
ATCC 19424											
*MIC*	<*0.016*				<*0.016*				<*0.016*		
ATCC 49226											
*MIC*	*0.032*				*0.032*				<*0.016*		

a N. gonorrhoeae *s*trains ATCC 19424 and ATCC 49226. PBP, penicillin-binding proteins.

b Concentration of β-lactam that inhibits 50% of Bocillin FL compared to that of a control containing no drug. Please see Fig. 2 for abbreviations.

c When none of the PBPs were inhibited using the regular concentrations, an extended range of 2 to 256 mg/L was used (MEC, AVI, SUL, REL, and TZ).

d MIC strips contain stable concentration gradient for piperacillin, ceftazidime, or ceftolozane (0.016 to 256 mg/L) in the presence of a fixed concentration of the BLI tazobactam or avibactam (4 mg/L).

Excluding imipenem, carbapenem MICs were 1.25- to 7.5-fold lower than the IC_50_s for PBP2 (Table S1), suggesting that inactivation of PBP2 at very low carbapenem concentrations causes considerable bacterial inhibition. Compared with ceftriaxone (same PBP2 IC_50_ = 0.01), a higher extent of PBP1 and PBP3 inactivation did not greatly enhance the effectivity of these compounds.

Third- and fourth-generation cephalosporins cefixime, cefotaxime, ceftazidime, cefepime, and ceftriaxone had the lowest IC_50_ values for PBP2 (0.01 to 0.02 mg/L) (Table 2), which were consistent between the two strains; however, a 2- to 4-fold MIC increase was observed for ATCC 49226. Second- and new-generation cephalosporins (cefoxitin, ceftaroline, and ceftolozane) were able to bind PBP2 at higher concentrations (IC_50_ = 0.21 to >2 mg/L). Cefoxitin inactivated the nonlethal target PBP3 with very low values in both strains (0.01 mg/L). Accordingly, the observed MIC values were 25- to 50-fold higher (0.25 and 0.5 mg/L) than the IC_50_s for this PBP (Table S1).

Aztreonam was selective for PBP2 in both strains (0.03 to 0.07 mg/L), but MIC values were 0.032 (ATCC 19424) and 0.125 mg/L (ATCC 49226). Amdinocillin, on the other hand, showed limited PBP2 inhibition (IC_50_ = 1.33 and 5.88 mg/L) and ranked the higher MIC values (1 and 8 mg/L) among β-lactam compounds.

Penicillins were selective for PBP2 in strain ATCC 19424 (0.01 to 0.19 mg/L), and with the sole exception of piperacillin, they caused little to no inactivation in strain ATCC 49226 (0.26 to >2 mg/L). While the PBP IC_50_s were consistent for both strains, the piperacillin MIC was substantially higher for strain ATCC 49226 (0.125 versus 0.001 mg/L, respectively).

Diazabicyclooctane (DBO) non-β-lactam β-lactamase inhibitors (avibactam and relebactam) were the only drugs showing PBP3 selective binding, while β-lactam-derived β-lactamase inhibitors (sulbactam and tazobactam) were selective for PBP2 (Table 2). The MICs of the DBO β-lactamase inhibitors (BLIs) were 55- to 400-fold higher than their IC_50_s for PBP3. Conversely, the MIC values for the β-lactam BLIs were 1.5- to 8-fold lower than their PBP2 IC_50_ values. Such remarkable difference was likely caused by the difference in target selectivity (PBP2 versus PBP3) between the β-lactamase inhibitors (Table S1).

**FIG 4:**
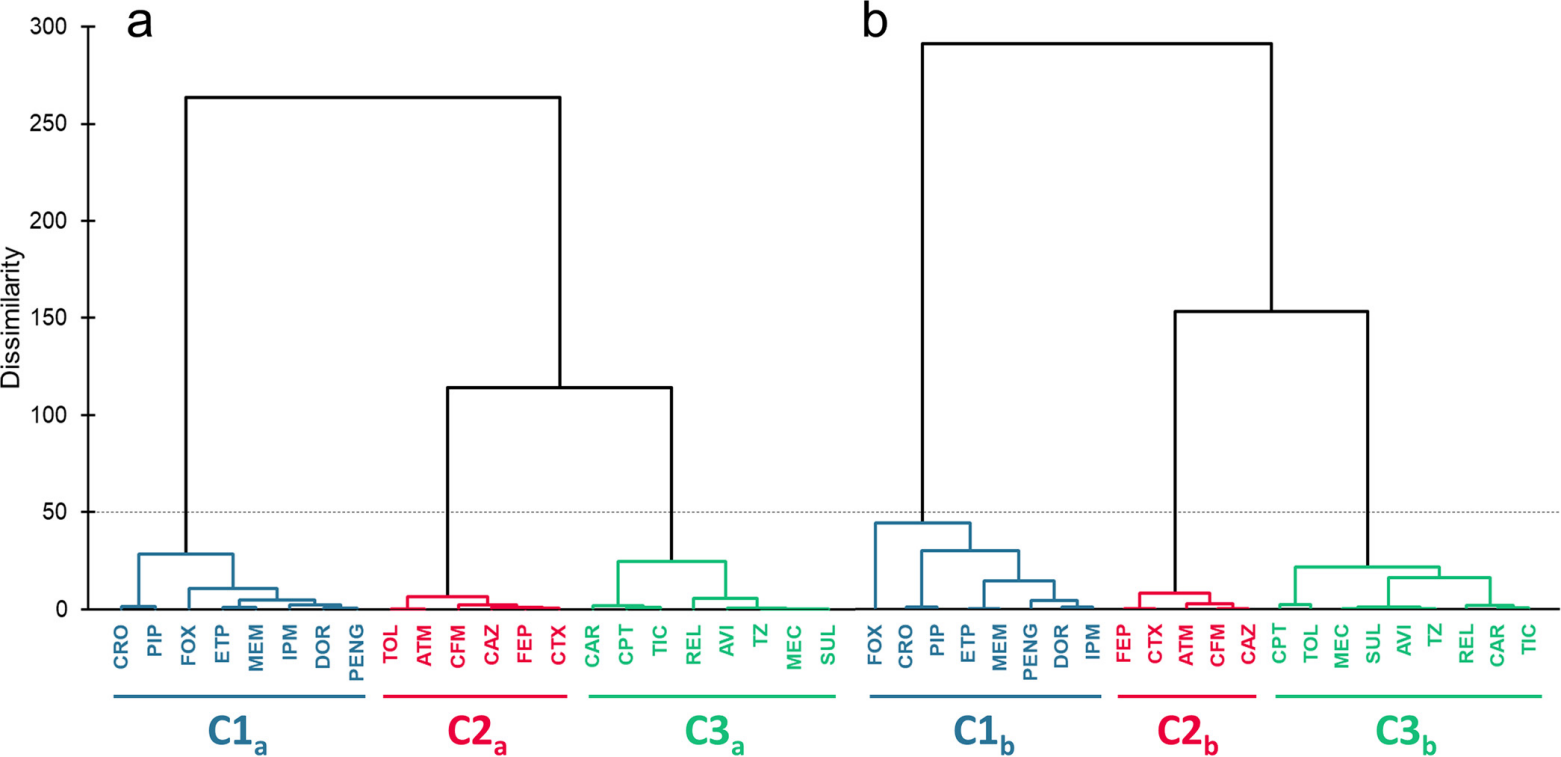
Agglomerative hierarchical clustering for logarithmic IC_50_ data of the tested 22 drugs with penicillin-binding proteins in isolated membranes of N. gonorrhoeae strains (a) ATCC 19424 and (b) ATCC 49226 using the XLSTAT program. C1 to C3 represent clusters 1 to 3. Please see Fig. 2 for abbreviations.

The principal-component analysis of the log-transformed PBP IC_50_s showed that the first two eigenvectors explained 89.9% of the total variance of the two strains for the 22 compounds (not shown). Agglomerative hierarchical clustering (AHC) identified the same three clusters with distinct PBP occupancy patterns in both strains (Fig. 4). The first cluster contained β-lactams that predominantly targeted PBP3 (0.01 to 0.11 mg/L), while also binding PBP2 at low concentrations in both strains (0.01 to 0.26 mg/L) (carbapenems, cefoxitin, ceftriaxone, penicillin G, and piperacillin). The second cluster comprised compounds that exclusively inactivated PBP2 (third-generation cephalosporins [but ceftriaxone] and aztreonam). Ceftolozane differed in the two analyses, belonging to cluster 3 in the ATCC 49226 strain. The third cluster included compounds that had PBP2, PBP3, or both as their primary targets but only at substantially higher concentrations (new-generation cephalosporins, imipenem, BLIs, carbenicillin, ticarcillin, and amdinocillin).

## DISCUSSION

With few preclinical candidates targeting MDR gonococci in the drug development pipeline and vaccine development being an unlikely solution in the short term due to high antigenic variability in clinical isolates, public health control of gonorrhea relies totally on appropriate antimicrobial treatment (2, 4, 10, 31).

The current emergence of resistance to first-line empirical gonococcal therapy (including ESCs) calls for the design and optimization of effective combination therapies (17, 27, 32, 33). The optimal first-line therapy should be widely available, highly effective (if possible as a single dose), lack toxicity, and with a microbiologic cure rate of >95% of patients (3). To gain insights into the molecular basis for optimizing drug combinations that maximize treatment efficacy and suppress resistance emergence, we characterized the occupancy patterns for 18 currently available and clinically relevant β-lactams and four β-lactamase inhibitors in N. gonorrhoeae.

Although no major differences were observed for the signal intensities of the three studied PBPs and PBP selectivity across both strains, remarkable differences were found for the binding IC_50_s and MIC values. In general, PBP profiles and occupancy patterns in the two studied strains were comparable to previously published data (40).

Carbapenems (except imipenem) were the only compounds that were coselective for PBP2 and PBP3 in both strains. Expectedly, compared with the first-line drug ceftriaxone, the increased PBP3 inhibition didn’t enhance bacterial growth inhibition (36, 41). In contrast to what has been observed in Enterobacterales and P. aeruginosa, imipenem showed the lowest PBP inhibition and the highest MIC values amid carbapenems (53, 54). Moreover, like doripenem, it is not likely to retain activity against cephalosporin-resistant N. gonorrhoeae isolates (55). In contrast, ertapenem and meropenem were 8 times more active and showed the lowest IC_50_s for PBP2 and 3 in both strains, mostly unaltered by the PBP2 type XXII and the *mtrR* mutation from strain ATCC 49226. Ertapenem and meropenem are the most effective drugs of this class, and potential candidates for possible N. gonorrhoeae alternative treatment for the ESC-resistant isolates. Furthermore, ertapenem could possibly be part of dual antimicrobial therapy (once a day) if the genetic resistance determinants are properly characterized (10, 14, 29).

As previously reported, third-generation cephalosporins cefixime, cefotaxime, and ceftazidime (acylation rates comparable to ceftriaxone) showed the greatest PBP2 inhibition in both strains. All of the drugs from this subclass showed a marginal 2-fold MIC increase except cefixime, which displayed a 4-fold higher MIC for strain ATCC 49226. Besides its epistatic nature, it seems that cefixime resistance may be more affected by *penA* and *mtrR* mutations compared to ceftriaxone, a possible explanation for the earlier therapeutic failures (16, 33, 42).

Cefoxitin inactivated PBP3 and PBP1 (slow PBP2 acylation rates). Although it could be a treatment option (plus probenecid) for penicillinase-producing isolates, there is not enough clinical evidence to support its efficacy against isolates with mutations in PBP2 or *mtrR* (33).

New-generation cephalosporins required the highest concentrations to inactivate PBP2 and demonstrated the lowest antibacterial activity in this subclass. Ceftaroline, with low IC_50_ values, exhibited reduced antimicrobial efficacy for strain ATCC 49226. The A39T missense mutation in *mtrR* and the PBP2 variant (type XXII) present in this strain may contribute to the 16-fold MIC increase (together with epistatic loci affecting antibiotic resistance) (16). Ceftolozane has better outer membrane permeability and stability toward class C beta-lactamases, leading to improved efficacy against P. aeruginosa. However, this feature limits PBP2 acylation capability and thus growth inhibition in N. gonorrhoeae (MIC = 0.25 mg/L) (33). A time of free drug above MIC (*f*T_>MIC_) of 20 to 24 h (longer for pharyngeal gonorrhea) needed for therapeutic efficacy, would rarely be achieved with ESCs with MICs ≥0.25 mg/L (42).

Aztreonam selectivity for PBP2 was markedly affected by the presence of the PBP2 type XXII and *mtrR* mutations. In a recent small clinical trial, a single 2-g aztreonam dose was an effective ESC-sparing alternative to treat gonococcal urethritis. However, in isolates with an MIC above 0.5 mg/L, it only achieved a 50% cure rate and was not a reliable treatment for pharyngeal infections (56). Amdinocillin, with limited PBP2 inhibition, has demonstrated poor therapeutic activity for isolates that are nonsusceptible to common first-line antigonococcal antimicrobials (30).

Like in previous reports for penicillins, modifications of the *penA* gene (type XXII) elicited a significant reduction of PBP2 binding, with the sole exception of piperacillin, with comparable IC_50_ values among the two strains. The observed 125-fold higher piperacillin MIC for strain ATCC 49226 suggested that reduced uptake (porin or altered efflux) could be the underlying mechanism for the MIC shift (17, 29). The *porB1a* allele has been associated with lower resistance levels compared to *porB1b*; however, the impact of the double mutation G120D/A121G on β-lactams resistance needs to be further explored (57 to 59). Furthermore, the A375T substitution in the *ponA* gene present in the strain ATCC 49226 has been identified in N. gonorrhoeae clinical isolates with high-level resistance to penicillins (37).

Since their introduction, continuous β-lactam development has allowed combinations of two different compounds that are not affected by the same resistance determinants (12, 60, 61). Several β-lactam-derived BLIs have been developed to treat infections by β-lactamase-producing organisms (clavulanate, sulbactam, and tazobactam) combined with β-lactams. Recently, two novel classes of non-β-lactam BLIs have been introduced to the clinic: the boronate-based (vaborbactam) and the diazabicyclooctanes (DBOs) (avibactam, relebactam, zidebactam, nacubactam, etc.). These compounds have shown improved spectrum or β-lactam enhancing activity (PBP2-binding), acting synergistically with the partner β-lactam (62). With the current low penicillinase-producing N. gonorrhoeae prevalence, combination therapies could focus on enhancing the partner activity (additive [PBP2] or complementary [PBP1] PBP-binding) (13).

As previously described in other Gram-negatives, novel BLIs (DBOs) showed neither an extensive PBP occupancy nor a significant increase or decrease of their primary target attainment in any of the studied strains (63). The potential therapeutic efficacy of sulbactam or tazobactam alone was better than that of the DBO-derived BLIs (avibactam and relebactam), against the two strains. When combined with their clinical partner β-lactam (piperacillin-tazobactam and ceftolozane-tazobactam), β-lactam BLIs displayed a notable increase of the antibacterial activity against both strains (4- to >16-fold MIC reduction). DBO-based β-lactamase inhibitor avibactam showed a modest ceftazidime MIC reduction. Neither of the studied strains expresses a β-lactamase, so the improved efficacy could rely on additive (PBP2; tazobactam) target binding (34, 62).

Clinically available combinations, piperacillin-tazobactam, ceftolozane-tazobactam, and to a lesser extent ceftazidime-avibactam, though unsuitable for empirical use (short half-lives and parenteral route of administration), could be considered for individual patients with ceftriaxone-resistant gonococcal infection once MICs (or preferably genetic point-of-care antimicrobial resistance tests) are available (34).

Dissimilar antimicrobial stewardship programs worldwide and the lack of clinical data and breakpoints to support the use of rationally improved antimicrobial therapies, challenge the treatment against N. gonorrhoeae (9, 42). Our study presents the first data set on PBP occupancy in N. gonorrhoeae for 22 clinically relevant β-lactams and BLIs. A limitation of our study is the use of two N. gonorrhoeae strains without a mosaic PBP2 allele and lack of studies on isogenic strains with knockout or overexpression of efflux, and porins. However, our comprehensive data set comprised 22 drugs studied at least in triplicate in two N. gonorrhoeae strains used for clinical control assessment procedures.

Future studies evaluating the target site penetration of β-lactams in reduced outer membrane permeability isolates and to systematically link PBP occupancy patterns to bacterial killing (especially in isolates producing mosaic PBP2 alleles) are warranted. We think that the present work provides relevant data to allow selecting β-lactams and combinations for future synergy studies with β-lactamase inhibitors and other antibiotic classes against resistant N. gonorrhoeae isolates.

Without a doubt, a coordinated effort among basic, translational, and clinical research is needed to develop *in vitro* and *in silico* pharmacokinetic/pharmacodynamics (PK/PD) models that evaluate and predict antibacterial activity and resistance emergence. Future mechanistically informed clinical trials evaluating rationally designed therapies could help advance the standard of care toward the much-needed individually tailored treatment.

## MATERIALS AND METHODS

### *In vitro* susceptibility testing.

The MIC of β-lactams and β-lactamase inhibitors (BLIs) was determined by the standard Clinical and Laboratory Standards Institute (CLSI) broth microdilution method (43). Imipenem was purchased from Fresenius Kabi (Barcelona, Spain); meropenem from Aurovitas (Madrid, Spain); ertapenem from Merck Sharp & Dohme (Haarlem, Netherlands); penicillin G from Laboratorio Reig Jofré SA (Barcelona, Spain); ceftaroline from Pfizer Pharmaceuticals (Ringsend, Ireland); cefotaxime, ceftriaxone, cefoxitin, and ceftazidime from Laboratorios Normon (Madrid, Spain); cefepime from Accord Healthcare (Barcelona, Spain); aztreonam from Bristol-Myers Squibb (Madrid, Spain); and doripenem, cefixime, ceftolozane, amdinocillin, carbenicillin, piperacillin, ticarcillin, avibactam, relebactam, sulbactam, and tazobactam from MedChem Express (Sollentuna, Sweden). Ceftazidime-avibactam and ceftolozane-tazobactam MICs were determined by MIC test strip from Liofilchem (Rosetto degli Abruzzi, Italy). MIC values were determined from at least three independent experiments.

### PBP-binding assays.

To enhance the strength of our PBP-binding (reported as the 50% inhibitory concentration; IC_50_) data sets and evaluate strain-to-strain variability, we studied two wild-type reference N. gonorrhoeae (NG) strains, ATCC 19424 and ATCC 49226, type strain and CLSI-recommended strain for quality control assessment procedures, respectively. The PBP-binding IC_50_s were determined in membrane preparations from the reference strains following previously described protocols (40).

N. gonorrhoeae cultures were grown in phosphate-buffered gonococcal medium (GCP) broth supplemented with 1/100 volume 4.2% sodium bicarbonate and 1/100 volume Kellogg’s supplement on a 37°C shaking incubator (200 rpm). Approximately 400 mL late-log phase (OD_600nm_ = 1) was collected by centrifugation, washed, and resuspended in 20 mM KH_2_PO_4_ with 140 mM NaCl pH 7.5. Cells were sonicated and centrifuged at 4,000 × *g* for 20 min. Bacterial membranes were collected by ultracentrifugation. Binding reactions were conducted for 22 chemically diverse β-lactams and β-lactamase inhibitors using 20 micrograms of membrane preparation proteins for 30 min at 37°C (range of concentrations tested = 0.0156 to 2 mg/L). When no measurable binding was observed for any of the PBPs, an upper or lower extended concentration range was used (0.001 to 0.125 or 2 to 256 mg/L when indicated). The initial incubation allows that each of the drugs binds to each of the PBP target receptors depending on its second-order acylation rate constant (*k_2_/K_s_*; inhibitory potency of a β-lactam) (44 to 46). After the initial incubation, membrane preparations were labeled with 25 μM Bocillin FL for 30 min (Bocillin FL is a fluorophore-conjugated penicillin V analog that binds to all PBPs). Unbound PBP molecules are then available to bind to Bocillin FL. For a given antibiotic concentration, the weaker the Bocillin FL signal, the greater the binding capacity for each of the PBPs (47). PBPs were separated on SDS-PAGE (Bio-Rad Laboratories, Hercules, CA), visualized using a Typhoon FLA 9500 biomolecular imager, and IC_50_s quantified using ImageQuantTL 8.1 (GE Healthcare Bio-Sciences AB, Björkgatan, 30 751 84 Uppsala). The amount of a PBP bound by a particular beta-lactam is determined by the remaining PBP molecules available to react with Bocillin FL, in comparison to those bound by Bocillin FL in the absence of the beta-lactam. Binding affinities were reported as the β-lactam or BLI concentrations that half-maximally inhibited Bocillin FL binding (IC_50_s) and were determined from at least three independent experiments.

### Data analysis.

Agglomerative hierarchical clustering (AHC) was performed in the XLSTAT software (v2021.5; Addinsoft). We analyzed the logarithmic PBP-binding data of twenty-two drugs in both N. gonorrhoeae strains to identify different clusters of the studied drugs based on their binding data. Agglomerative hierarchical clustering was used based on the Euclidean distance for calculating the degree of dissimilarity. The Ward's method was used for agglomeration to generate the dendrogram, and three differentiated clusters were observed in each strain.
