# Supplementary material for: Penicillin-Binding Protein Occupancy Dataset for 18 β-Lactams and 4 β-Lactamase Inhibitors in Neisseria gonorrhoeae
Source: Microbiol Spectr. 2023 Apr 24;11(3):e00692-23. doi: 10.1128/spectrum.00692-23 (PMC10269775; doi:10.1128/spectrum.00692-23)
Supplement: Supplemental file 1 — Fig. S1 and S2 and Table S1. Download spectrum.00692-23-s0001.pdf, PDF file, 0.4 MB [file spectrum.00692-23-s0001.pdf]

Supplementary data for

López-Argüello et al; Penicillin-binding protein occupancy dataset for 18 β-lactams and 4 β-lactamase inhibitors in *Neisseria gonorrhoeae*

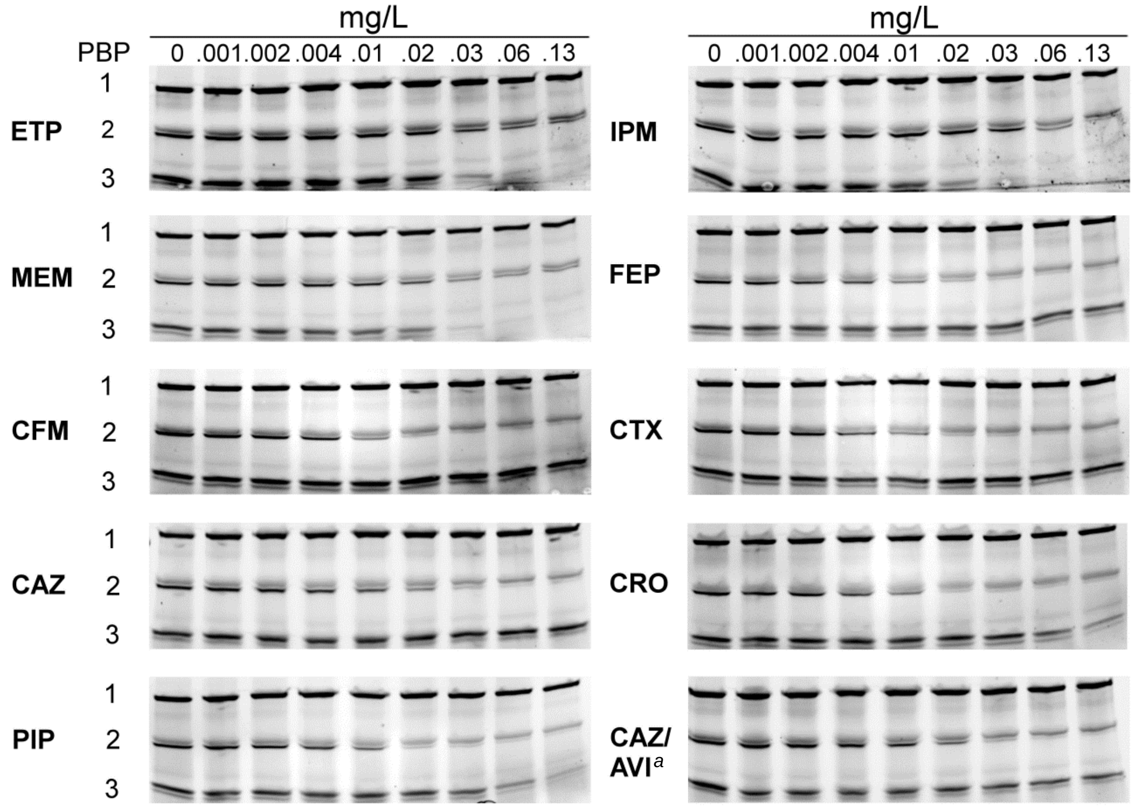

Figure S1. Binding patterns of β-lactams for *N. gonorrhoeae* PBPs from strain ATCC 19424. Range of concentrations = 0.001 to 0.125 mg/L. ETP, ertapenem; IPM, imipenem; MEM, meropenem; FEP, cefepime; CFM, cefixime; CTX, cefotaxime; CAZ, ceftazidime; CRO, ceftriaxone; PIP, piperacillin; CAZ/AVI, ceftazidime/avibactam. The membrane preparations were incubated with the indicated β-lactams for 30 min before Bocillin FL labeling. Labeled PBPs were separated by SDS-PAGE and detected using a fluorimager.[a] CAZ/AVI IC_{50} was determined in the presence of a fixed concentration of avibactam (4 mg/L).

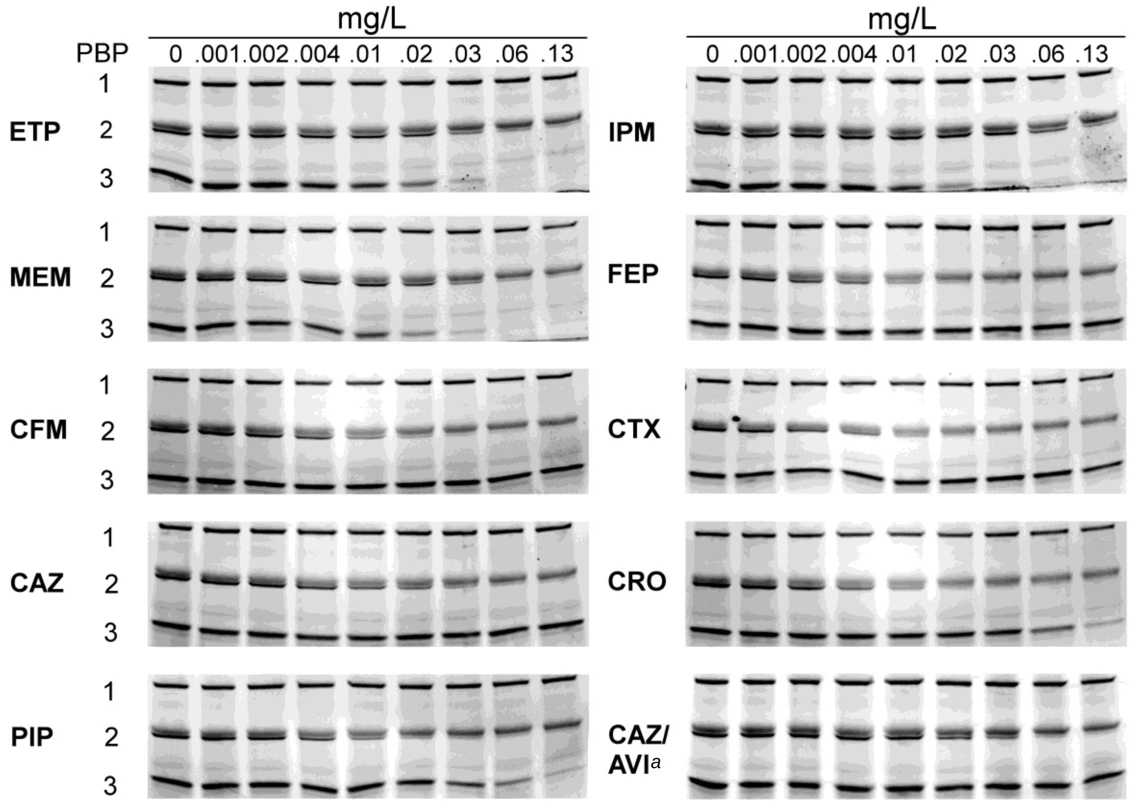

Figure S2. Binding patterns of β-lactams for *N. gonorrhoeae* PBPs from strain ATCC 49226. Range of concentrations = 0.001 to 0.125 mg/L. Please see Figure S1 for abbreviations. The membrane preparations were incubated with the indicated β-lactams for 30 min before Bocillin FL labeling. Labeled PBPs were separated by SDS-PAGE and detected using a fluorimager. [a] CAZ/AVI IC_{50} was determined in the presence of a fixed concentration of avibactam (4 mg/L).

Table S1. PBP IC_{50}, MICs and target selectivity of β-lactam antibiotics and BLIs in *N. gonorrhoeae* ATCC 19424 and ATCC 49226.

ATCC 19424	IC_{50}[a]			MIC (mg/L)	min. IC_{50}[a]	MIC/ min. IC_{50}[b]	Target Selectivity[c]
**Drug**	**PBP1**	**PBP2**	**PBP3**				
**DOR**	1.13	**0.01**	**0.02**	0.004	0.01	0.40	**PBP2 and 3**
**ETP**	0.48	**0.03**	**0.02**	0.004	0.02	0.20	**PBP2 and 3**
**IPM**	0.4	0.07	**0.01**	0.032	0.01	3.20	**PBP3**
**MEM**	0.24	**0.04**	**0.03**	0.008	0.03	0.27	**PBP2 and 3**
**FEP**	> 2	**0.01**	1.57	0.008	0.01	0.80	**PBP2**
**CFM**	0.92	**0.01**	> 2	0.004	0.01	0.40	**PBP2**
**CTX**	1.85	**0.01**	1.43	0.004	0.01	0.40	**PBP2**
**FOX**	0.58	0.39	**0.01**	0.25	0.01	25.00	**PBP3**
**CPT**	> 2	**0.15**	1.9	0.032	0.15	0.21	**PBP2**
**CAZ**	> 2	**0.01**	> 2	0.016	0.01	1.60	**PBP2**
**TOL**	> 2	**0.04**	> 2	0.032	0.04	0.80	**PBP2**
**CRO**	1.87	**0.01**	0.07	0.004	0.01	0.40	**PBP2**
**ATM**	> 2	**0.03**	> 2	0.032	0.03	1.07	**PBP2**
**MEC**	102.32	**1.33**	11.92	1	1.33	0.75	**PBP2**
**CAR**	> 2	**0.13**	0.64	0.032	0.13	0.25	**PBP2**
**PenG**	1.89	**0.05**	**0.02**	0.063	0.02	3.15	**PBP2 and 3**
**PIP**	> 2	**0.02**	0.11	0.001	0.02	0.05	**PBP2**
**TIC**	> 2	0.19	1.05	0.016	0.19	0.08	**PBP2**
**AVI**	> 512	117.15	**2.33**	128	2.33	54.94	**PBP3**
**REL**	> 512	512	**1.27**	512	1.27	403.15	**PBP3**
**SUL**	55.42	**1.18**	9.31	0.25	1.18	0.21	**PBP2**
**TZ**	19.21	**1.07**	4.72	0.125	1.07	0.12	**PBP2**

Table S1. PBP IC_{50}, MICs and target selectivity of β-lactam antibiotics and BLIs in *N. gonorrhoeae* ATCC 19424 and ATCC 49226 - **continued**

**ATCC 49226**	**IC_{50}[a]**			**MIC (mg/L)**	**min. IC_{50}[b]**	**MIC/ min. IC_{50}[c]**	**Target Selectivity[a]**
**Drug**	**PBP1**	**PBP2**	**PBP3**				
**DOR**	1.08	**0.09**	**0.02**	0.032	0.02	2.13	**PBP2 and 3**
**ETP**	0.57	**0.01**	**0.01**	0.008	0.01	0.80	**PBP2 and 3**
**IPM**	0.8	0.17	**0.01**	0.064	0.01	6.40	**PBP3**
**MEM**	0.45	**0.02**	**0.01**	0.016	0.01	1.60	**PBP2 and 3**
**FEP**	> 2	**0.01**	1.18	0.032	0.01	3.20	**PBP2**
**CFM**	> 2	**0.02**	> 2	0.016	0.02	0.80	**PBP2**
**CTX**	> 2	**0.01**	1.02	0.008	0.01	0.80	**PBP2**
**FOX**	0.91	> 2	**0.01**	0.5	0.01	50.00	**PBP3**
**CPT**	> 2	**0.21**	1.29	0.5	0.21	2.37	**PBP2**
**CAZ**	> 2	**0.01**	> 2	0.032	0.01	3.20	**PBP2**
**TOL**	> 2	**0.27**	> 2	0.25	0.27	0.93	**PBP2**
**CRO**	1.9	**0.01**	0.07	0.008	0.01	0.80	**PBP2**
**ATM**	> 2	**0.07**	> 2	0.25	0.07	3.57	**PBP2**
**MEC**	76.59	**2.94**	4.62	8	2.94	2.72	**PBP2**
**CAR**	> 2	> 2	**0.35**	0.5	0.35	1.43	**PBP3**
**PenG**	> 2	0.26	**0.01**	0.125	0.01	12.50	**PBP3**
**PIP**	> 2	**0.01**	0.09	0.125	0.01	12.50	**PBP2**
**TIC**	> 2	> 2	0.63	0.5	0.63	0.79	**PBP3**
**AVI**	> 512	> 512	**5.40**	512	5.40	94.81	**PBP3**
**REL**	> 512	> 512	**1.31**	512	1.31	390.84	**PBP3**
**SUL**	203.13	**6.02**	**6.36**	4	6.02	0.66	**PBP2 and 3**
**TZ**	25.11	**1.44**	5.69	1	1.44	0.69	**PBP2**

[a] IC_{50} represents the concentration that half-maximally inhibits each of the PBP targets. Bold means the selectivity of each drug. A compound is considered selective for a PBP if its IC_{50} is at least 4-fold lower than that of the next most inhibited PBP. When a second PBP falls below the 4-fold threshold, the compound is considered to be coselective for two PBPs. DOR, doripenem; ETP, ertapenem; IPM, imipenem; MEM, meropenem; FEP, cefepime; CFM, cefixime; CTX, cefotaxime; FOX, cefoxitin; CPT, ceftaroline; CAZ, ceftazidime; TOL, ceftolozane; CRO, ceftriaxone; ATM, aztreonam; MEC, mecillinam (amdinocillin); CAR, carbenicillin; PenG, penicillin G; PIP, piperacillin; TIC, ticarcillin; AVI, avibactam; REL, relebactam; SUL, sulbactam; TZ, tazobactam.

[b] Minimum observed IC_{50} value.

[c] Ratio between the MIC and the minimum IC_{50} value. Higher ratios indicate lower antibacterial potency and may correlate with a lower therapeutic efficacy. Drugs with minimum IC_{50} values for PBP3 displayed the higher MIC values.
